# Disease Biomarkers of Giardiasis

**DOI:** 10.1155/2022/1932518

**Published:** 2022-08-26

**Authors:** Norhamizah Roshidi, Norsyahida Arifin

**Affiliations:** Institute for Research in Molecular Medicine, Universiti Sains Malaysia, 11800 Pulau Pinang, Malaysia

## Abstract

Giardiasis is a common, treatable intestinal disease that adversely affects underprivileged communities living in unsanitary conditions. Giardiasis causes a wide spectrum of gastrointestinal diseases in those infected, ranging from subclinical disease that can manifest as irritable bowel syndrome with persistent abdominal symptoms. Importantly, giardiasis has been identified as a predictor of malnutrition among young children in rural areas and as a cause of waterborne mass epidemics endangering not only humans but also animals in a broad clinical, social, and economic spectrum. While the diagnosis of giardiasis is heavily dependent on the presence of cysts and/or trophozoites detected using microscopy, the intermittent cyst excretion, low infection intensity, and low sensitivity method m4akes fecal examination unrewarding, thus urging the need for an improved diagnostic method for giardiasis. Proteins are key compounds in biosynthesis, cells, tissues, and organ signaling, carrying important information related to biological and pathogenic processes, as well as pharmacological responses to therapeutic intervention, and are therefore important indicators for determining disease onset, progression, and drug treatment effectiveness. In connection with this, proteins could serve as promising biomarkers for antigen-antibody detection, as well as vaccine candidates. This article is aimed at providing a comprehensive overview of proteins, serological, molecular, inflammatory, volatile, and hormonal biomarkers associated with giardiasis and their potential for diagnostics and therapeutics.

## 1. Introduction

Giardiasis is one of the most common causes of diarrheal infection worldwide due to infections by the etiologic agent *Giardia duodenalis* (synonym of *G. intestinalis* and *G. lamblia*). Despite being treatable and self-limiting, concern remains, as approximately 280 million giardiasis cases are reported annually, partly contributing to a total of 1.6 million cases of diarrheal deaths reported in 2016 [1]. Significantly, one in nine child deaths are due to diarrhea, which contributes to 2,195 deaths per day, bringing the total to 801,000 child deaths every year—more than the combined cases of malaria, measles, and AIDS—hence placing diarrhea as an important threat on the global health radar [2]. This figure is particularly disturbing given that diarrheal diseases account for over 20% of all deaths in young children in poor countries, as compared to fewer than 1% in the more economically developed countries [3]. With diarrhea as the second leading cause of infectious disease-related morbidity and mortality after pneumonia among children, it is important to control the progression of giardiasis to reduce the likelihood of an increase in diarrhea-related deaths especially in extreme cases of diarrhea in infants and malnourished children [4].

Giardiasis is present in human and animal environments and is thus of significant clinical and economic importance not only to humans but also to the environment, livestock, and pet animals, which demand One Health's integrated approach to control giardiasis comprehensively. The last few decades have witnessed *Giardia* being elevated from its place as a commensal to that of an important pathogen, leading to its inclusion in the WHO Neglected Diseases Initiative in 2004. Consequently, giardiasis has received significant attention from researchers, especially regarding its diagnostics and therapeutics, as well as the fundamental understanding of the immunological mechanisms, disease manifestations and pathophysiology, parasitic virulence characteristics, and the determinants of the hosts' responses, which are not yet clearly defined.

The treatment of giardiasis depends on the administration of drugs from six classes of compounds, namely, 5-nitroimidazoles (5-NIs), benzimidazole (BI) derivatives, quinacrine, furazolidone, paromomycin, and nitazoxanide. The most common drugs given to patients, however, are the 5-NI compounds such as metronidazole, tinidazole, ornidazole, and secnidazole [5, 6]. Metronidazole is usually given in three divided oral doses of 250 mg daily for 5–10 days with a reported efficacy of 80 to 95%. Meanwhile, tinidazole has recently become the FDA-approved drug of choice for giardiasis in the US due to its high efficacy (about 90%), tolerability, and convenience because only a single oral dose is required. Several BIs such as albendazole and mebendazole have also been reported to be effective and are often used as anti-Giardiasis drugs not only in humans but also in pets and livestock [7].

Over the years, the diagnosis of giardiasis has become diversified and has progressed, exhibiting the incorporation of molecular and immunological-based assays in a series of direct and indirect test panels. Early and accurate diagnosis is very important for the treatment and prevention of giardiasis, to prevent disease progression and to reduce disease burden. While a human vaccine against giardiasis is still not available, diagnosis has improved with the support of the availability of many well-established commercial tests on the market, although it is relatively costly for use in underdeveloped countries where giardiasis is prevalent. Laboratory diagnosis of giardiasis relies on microscopic identification of cysts and trophozoites in fecal samples, and this is considered a controversial gold standard method due to several limiting factors that influence the sensitivity of microscopy techniques, such as the selection of direct or concentration methods, the number of fecal samples examined, and the expertise of professionally trained technicians, in addition to the intermittent nature of cyst excretion [8].

In the new era of precision medicine and predictive diagnostics, there is a need to reexamine the existing and new discoveries that can be developed and potentially translated into new technologies in the quest to provide accurate diagnosis and effective treatment to patients. In this view, biomarkers are key options that can be manipulated to be applied into advanced prognostics and diagnostics, with the aim of providing better disease diagnosis. Biomarkers are biological characteristics that can be objectively measured in biofluid and tissue samples and are defined as indicators of a physiological as well as a pathological process or pharmacological response to a therapeutic intervention [9]. Conceptually, biomarkers can be of diagnostic, prognostic, or therapeutic value, with several classifications depending on type, characteristics, application, genetics, and molecular biology methods as well as their properties [10]. The up- and downregulation of biomarkers in response to a change in the body is indicative of infection and can be used to determine disease onset, progression, and patient susceptibility to develop a certain type of disease or to predict the efficacy of treatment at a particular disease stage [11]. Given that survival rates are highly dependent on early diagnosis and the administration of effective treatments, the detection of disease biomarkers can aid clinical decision-making, permit faster treatment, and ultimately inform therapeutic interventions to enhance patient survival and quality of life. The exploration of biomarker assays has evolved over time, in parallel to the context of use of biomarkers, which has also undergone such evolution. In recent years, a great deal of research has been conducted to identify novel biomarkers through multiple approaches that combine medical, analytical chemistry, and bioinformatics. Therefore, the search for specific biomarkers becomes crucial by virtue of the need for improved diagnosis and therapeutics for giardiasis—the aim that we intended to highlight in this article.

## 2. Protein Biomarkers

Several immunodiagnostics assays have been long developed and used for giardiasis diagnosis, either to detect antibodies, such as ELISA, or to detect *Giardia* fecal antigens in fresh or formalin-preserved fecal specimens, such as the rapid antigen detection test (RDT), the nonenzymatic immunochromatographic, and immunofluorescence antibody test (IFAT). The identification of *Giardia* antigens is challenged by the occurrence of antigenic variation and the different antigenic profiles of isolates from different geographical areas. Nevertheless, fecal antigen detection is preferred, as serologic testing has been proven to be of little value in giardiasis diagnosis because of the biological characteristics of the parasite, the lack of suitable antigens, and the fact that the long-term humoral immune response after a natural giardiasis infection is not well understood [12, 13]. Thus, few antibody detection kits have been commercialized in the market, but there are numerous commercial antigen detection kits for giardiasis, with some displaying sensitivities and specificities of 100% [14, 15]. In fact, tests have been produced for the simultaneous detection of intestinal pathogens, namely, *Giardia* and *Cryptosporidium* or *Giardia* spp., *Cryptosporidium*, and *Entamoeba* species antigens in fecal specimens, allowing effective treatment of patients with enteric diseases even if pathogenic species cannot be distinguished [16]. Although these tests are considered better than microscopy in terms of sensitivity and specificity, they should not replace the use of conventional parasitological analysis but instead should be used as complementary tests, especially for patients with negative microscopy results but with persistent symptoms.

Proteins are key compounds in biosynthesis and cell, tissue, and organ signaling and are crucial in providing structural stability in the cells and tissues of living organisms. From a diagnostic perspective, protein biomarkers are particularly popular due to the availability of a large range of analytical instrumentation, which can identify and quantify proteins in complex biological samples. Much of the previous literature has described the identification of *Giardia* major native proteins as target antigens with various functions and localizations, namely variant surface proteins (VSPs), giardins, tubulins (cytoskeletal proteins), heat-shock proteins (HSPs), cyst wall proteins (CWPs) and metabolic-proteins such as enolase-a, fructose-1,6-biphosphate aldose (FAB), arginine deaminase (ADI), and ornithine carbamoyl transferase (OCT) [11]. Variant-specific surface proteins (VSPs) are cysteine-rich proteins found on the surface of the trophozoites; they are characterized as having many CXXC motifs as well as a highly conserved C-terminus CRGKA tail, and their molecular masses are between 20 and 200 kDa. In addition to being involved in immune evasion and host-parasite interaction, VSPs are also components of cellular signaling. Prucca and Lujan published an extensive review on VSPs, discussing the antigenic variation, the mechanism, and the genomic organization of VSPs [17]. The main feature of VSPs is that they undergo surface antigenic variation every six to 13 generations to escape the host humoral immune response through on-off switching of the expression genes encoding VSPs, a phenomenon that is regulated by a mechanism controlled by interfering RNA (iRNA). While this phenomenon of antigenic variation raises questions about the value of VSPs as diagnostic and immunological targets [18], several VSPs have still been reported as being highly immunogenic proteins that activate the humoral immune response. The most characterized VSP is VSPH7, a 56 kDa protein that is considered highly immunogenic, in addition to VSP 5G8 which induces a strong humoral immune response when injected into mice, suggesting its potential as a candidate for vaccine development [19, 20]. Additionally, two recombinant proteins of VSP3 and VSP5, originating from assemblages A and B, respectively, were expressed in *E. coli* and showed good reactivity to IgM, IgA, and IgG when tested in multiplex bead immunoassay [12].

Giardins, on the other hand, are small, structural, constitutive proteins (29–38 kDa) that are components of the Giardia cytoskeleton within the ventral disc, and they can be classified into four groups, namely alpha- (*α*-) giardin, beta- (*β*-) giardin, gamma- (*γ*-) giardin, and delta- (*δ*-) giardin. The *α*-giardins form a large class of Annexin-like proteins located at the outer edges of the ventral disc microribbons, whereas *β*-giardins are striated fibre-assemblin-like proteins and are closely associated with microtubules. Gamma- (*γ*-) giardins also have been identified as microribbon proteins; however, their localization and functions within the ventral disc microribbon are still uncertain [21, 22]. Delta- (*δ*-) giardins, on the other hand, are different from *α*-giardin and *γ*-giardin but share conserved AA motifs with *β*-giardin, suggesting that they belong to the same protein family [22, 23]. Together, these proteins are associated with the plasma membrane and membrane systems and participate in the movement of the cytoskeleton and signal transduction in the cell, regulating the growth and proliferation of cells, and they also participate in the encystation and excystation process of *Giardia* cysts [24]. A study by Palm et al. detected a 32 kDa highly immunoreactive protein in sera from acute patients with giardiasis, and this protein was later identified as *α*-1-giardin [22]. Meanwhile, Weiland et al. identified 14 coding genes for *α*-giardins (*α*-4 to *α*-6, *α*-8 to *α*-13, and *α*-15 to *α*-19) in *G. lamblia* [25]. Characterization of *α*-1 giardins further indicates that the immunoreactive region of this protein is located between amino acids 160 and 200, through an epitope that is also shared by the newly identified *α*-7.1 giardin, which is also a highly immunoreactive protein during human giardiasis [25]. Further studies have demonstrated that *α*-1-giardin not only stimulates the production of anti-*Giardia* antibodies (IgA and IgG2a) but also establishes protection against posterior challenges [11]. In addition, not only has the potential use of *α*-1-giardin been reported as a diagnostic biomarker in several commercial RDTs, such as the Triage Parasite Panel (BioSite Diagnostics, USA) but also additional findings on conserved amino acid and immunological cross-reactivity of various *Giardia* isolates support the continued development of *α*-1-giardin as an antigenic candidate for a vaccine against giardiasis [26–29].

An often-used strategy for the large-scale, efficient, and consistent production of protein is through recombinant technology utilizing various exogenous host systems: that is, bacterial, insect cells, yeast, or mammalian cells. Recombinantly produced proteins have two main advantages over native proteins: (i) recombinant protein can be consistently produced in a controlled environment at the desired amount, and (ii) a high throughput system can lower the production cost. Today, the production of recombinant proteins has become the core catalyst in the diagnostics and therapeutic industry segment with the emergence of various diagnostic tests for various diseases.

In a comprehensive proteomic study by Palm et al., 16 immunogenic proteins reactive towards acute patients' sera have been identified, some of which are variable surface proteins, *α*-giardins, arginine deiminase, ornithine carbamoyl transferase, and fructose-1,6-bisphosphate aldolase, of which six are novel (SALP-1, GTA-1, GTA-2, UPL-1, *α*-7.1-giardin, and *α*-7.3-giardin) [22]. Following this, the group has produced the recombinant form of *α*-1-, *α*-2-, and *α*-7.1-giardin, ornithine carbamoyl transferase (OCT), and arginine deiminase (ADI) proteins, which were found to be highly immunoreactive against serum samples from *G. duodenalis*-infected patients and presumed to be indicative of acute giardiasis. Meanwhile, a recombinant form of *α*-13 giardin was produced by Yu et al., yielding a protein with molecular weight of 40 kDa localized in the cytoplasm of *G. lamblia* trophozoites, suggesting that it is a cytoplasm-associated protein [24]. Importantly, the anti-*α*-13-giardin polyclonal antibody possesses good antigenic specificity as well as excellent binding activity with recombinant *α*-13-giardin and is hence an interesting target antigen to be used in the development of new methods for the diagnosis of giardiasis.

Another major functional protein of *Giardia* that forms the basic components of the cytoskeleton is tubulin. Tubulins exist as multiple isoforms with pIs of 4–5.5 and molecular weights of 54–58 kDa and have been visualized in the flagella, ventral disk, funis, and median body when fixed in formalin, whereas unfixed tubules showed different antigenic structures [13]. Microtubules are formed from the interaction between the polymerization of the tubulin isoforms of *α*- and *β*-tubulin monomers (heterodimers) with microtubule-associated proteins. The microtubules are believed to be the target site of two significant groups of benzimidazoles (BZs) and dinitroanilines [30, 31]. Meanwhile, the study by Campanati et al. proved that *Giardia* tubulin reacts with antibodies raised against very distinct immunogens [31]. Previous studies seeking to determine tubulin antigenicity have shown that there are at least five isoelectric variants of *G. duodenalis* tubulin that may represent primary targets for the immune system, since they are found in many organelles [32]. Although *α*- and *β*-tubulin are among the major proteins identified in acute giardiasis sera and are suggested to be among the immunodominant proteins in *Giardia*, the value of tubulin for diagnostics is still controversial due to its lack of specificity [22].

In response to challenges such as rapid change in temperature, pH, or other stressful treatment, mammalian, bacterial, protozoan, helminth, and even plant cells produce heat shock proteins (Hsp). *Giardia* trophozoites live in the intestine, a habitat where stresses are likely to occur due to the highly acidic conditions, and Hsp have been detected on the surface membrane of trophozoites, whereby they help the cells to survive this stress. In a study investigating temperature-related stress, it was found that the synthesis of [35S] methionine-labeled proteins of 30, 70, 83, and 100 kDa were increased with an increased temperature of 43°C, hence demonstrating the association between temperature and stress-related proteins [33]. The value of Hsp as a diagnostic biomarker has been demonstrated in several studies. For example, Al-Madani and AL-Khuzaie compared the concentration of Hsp70 in fecal samples of patients and nonpatients with giardiasis and showed that Hsp70 was present at a concentration sevenfold higher in the former than the latter (28.04 ng/mL versus 3.98 ng/mL), thus confirming that Hsp70 was significantly present in fecal matter of patients infected with *Giardia* parasites [34]. Further, Hsp90 is being studied as a drug target for several parasitic infections. The development of Hsp90 inhibitors as therapeutic agents has received significant interest, not only for giardiasis but also for malaria and amebiasis. In a study conducted by Debnath et al., it was demonstrated that Hsp90 is a viable target for giardiasis and amebiasis [35]. Studies also revealed that Hsp90 plays a role in inducing the encystation process when in the preencystation stage, and when it is combined with Hsp70, both proteins are thought to be important players in the differentiation process of *Giardia* [36].

Cyst wall proteins are expressed during the process of encystation as well as during the lifetime of a cyst; they are composed of Leu-rich repeats and a C-terminal Cys-rich region and can be classified into two different groups. Group I proteins are expressed during the early stages of encystation and are localized to encystation-specific vesicles (ESVs), whereas group II proteins are localized exclusively into the cyst wall surface. The two most interesting cyst wall proteins are the CWP1 and CWP2 proteins found in the cyst cell wall. They are closely related in terms of primary structure but differ by a 121-residue carboxyl-terminal extension. Lujan et al. previously identified a 26 kDa cyst wall protein, CWP1, and proved that this protein, when combined with a novel 39-kDa cyst wall protein (CWP2), forms a 65 kDa CWP1-CWP2 stable heterodimer complex before incorporation into the cyst wall [37]. Data from several studies recognized CWP1 as the target for diagnostic monoclonal antibodies to *Giardia* in clinical specimens, and the anti-CWP1 antibodies were found to reduce excystation of *Giardia* in vitro. Meanwhile, studies on CWP2, which has an additional positively charged domain at its C-terminus, have been shown to induce a host-immune response by the production of anti-*Giardia* IgA and IgG2a in mice immunized with CWP2 and simultaneously reduce cyst formation [38] [39]. In fact, the stimulation of the immune response induced by rCWP2 immunization has been found to be comparable to live infection with *G. muris* cysts in which the anti-rCWP2 immunoglobulin A (IgA) antibodies were detected in the feces and serum of the immunized mice, whereas anti-rCWP2 IgG1 and IgG2a antibodies were detected only in serum. In addition to this, mRNAs encoding for Th1 and Th2 cytokines were also detected in spleen and Peyer's patch cells from the immunized mice, coupled with the discovery of a low number of cysts in mice vaccinated with live cysts. Therefore, the researchers concluded that rCWP2 is a potential candidate antigen for the development of transmission-blocking vaccines [38].

Another important *Giardia* antigen is GSA 65, a glycoprotein of *M*_r_ 65,000 that is abundantly present in trophozoites and cysts and is immunologically detectable from the fecal matter of giardiasis patients. The first report on the isolation of GSA 65 was published by Rosoff et al. in 1986 [40]. Subsequently, Faubert reported that ELISA-GSA65 can detect giardiasis in at least 30% more cases than microscopy examination, with sensitivity and specificity ranging from 95% to 100% [13]. The ability of GSA 65 to maintain its antigenic structure under a wide variety of conditions makes it an ideal antigen in designing a sensitive immunodiagnostic assay for giardiasis, for which GSA 65 has been commercially used as a detector antigen in the Alexon ProSpecT/*Giardia* diagnostic test (Alexon, Inc., Mountain View, Calif.) [41].

## 3. Serological and Inflammatory Biomarkers

Symptomatic individuals have elevated circulating IgG, IgM, and IgA antibodies in response to cyst and trophozoite antigens which can be found in serum, saliva, milk, and duodenal biopsies [13]. *Giardia*-specific IgM antibodies are seen in the serum and gut mucosa approximately ten days after infection, and IgG and IgA are raised approximately one week later, indicating that *Giardia* antibodies might be recognized early in an infection.

Smith et al. detected specific IgG antibody in 81% of symptomatic cases and 12% of controls, and the secretion remained detectable for up to 18 months in most cases following drug treatment [42]. While IgG indicates an established infection, the IgM antibody level reduces to control levels between two and three weeks after drug treatment, indicating that IgM antibody might be a useful indicator of posttreatment [43]. Meanwhile, a study by El-Gebaly et al. found that the salivary and serum IgA and IgG responses against *G. duodenalis* infection were significantly higher in *Giardia* parasitized than non-*Giardia* parasitized children [44]. The study reported that the mean OD of salivary IgA in *Giardia* parasitized children was 0.424 and 0.282 for non-*Giardia*-parasitized children meanwhile the mean OD of serum IgA in *Giardia*-parasitized children was 0.491 and 0.364 for non-*Giardia* parasitized children. This may be explained by the fact that *Giardia* spp. induce strong production of secretory IgA (sIgA) antibody in humans as well as in animals, and this plays an important role in the protection and homeostatic regulation of intestinal mucosal epithelia, hence the clearance of the parasite [44]. Rodríguez et al. suggest that secretory anti-*Giardia* IgA levels measured in saliva samples may reflect local intestinal IgA responses elicited by *G. duodenalis* and that the determination of the level of slgA anti-*Giardia* could be a useful diagnostic tool for giardiasis diagnosis [45]. When used in IFAT and ELISA, the detection efficacy of both IgG and IgA is evident and comparable. However, IgM antibodies were almost undetectable in western blot analysis even when serum of patients with higher circulating antibody titers was used. This situation is improved when purified intact *Giardia* trophozoite proteins are used as antigens, and not the extract [46]. Nevertheless, the practicality of serological testing as a first-tier diagnostic test for giardiasis is debated because of the different antigenic identities of *Giardia* species from different geographical isolates, besides being difficult to differentiate between symptomatic and asymptomatic patients.

In saliva and milk samples, anti-*Giardia* antibodies were found to be present in around 52% and 59%, respectively, with a mean sIgA content 50 times higher in milk than in saliva [47]. Western blot analysis showed that there were 16 different *Giardia* proteins in the molecular weight region of 20–165 kDa reactive to anti-*Giardia* sIgA from these samples and that the major immunoreactive proteins were like the immunoreactive proteins identified by serum from acute giardiasis patients in a non-endemic country. Although milk sIgA recognized recombinant *Giardia* proteins such as alpha-1 giardin, ornithine carbamoyl transferase, VSP-4EX, arginine deaminase, and alpha-enolase, its reactivity to variant surface proteins (VSPs) was the strongest. These findings suggest that these antigens will be important targets in the development of new immunodiagnostic tools and vaccines for giardiasis [47].

Further, studies in an animal model have shown the production of cytokines, namely, IL-2, IL-4, IL-10, IL-13, IL-17, IL-22, TNF-A, and IFN-G p.i., in both adaptive and innate immune responses with giardiasis. However, previous research findings on the role of interleukins as disease biomarker for giardiasis have been inconsistent and contradictory. While several researchers observed a high level of IL-2, IL-4, and IL-10 in the infected group as compared to the control group [48–51], there are reports that state the level of IL-4 was decreased in patients with giardiasis [52]. Although the detection of cytokines presents an opportunity to measure the onset of giardiasis infections, however, cytokines are not capable of identifying a specific causative agent; rather, they are more generic biomarkers of infection [53].

Other than that, there is also a relatively small body of literature that is concerned with the immunological role of platelets against giardiasis. P-selectin is the essential protein of the selectin family of cell adhesion receptors expressed by platelets which helps in initial adhesion and rolling of platelets and leukocytes to areas of injury and inflammation. The study by Al-Hadraawy et al. revealed that the concentration of P-selectin is significantly increased in serum of patients infected with *G. lamblia* compared to the control group [54]. A possible reason to this may be due to the host response to giardiasis infection which results in increased expression of cell adhesion molecules to achieve its role in the uptake of effector cells to the site of infection. However, the lack of studies on P-selectin makes it difficult to justify the potential of P-selectin as a diagnostic biomarker of giardiasis; therefore, more in-depth studies are warranted.

Another host-specific protein studied was calprotectin, a calcium and zinc-binding protein which is an essential marker in inflammatory bowel disease. Calprotectin is a heterodimer of two calcium-binding proteins found in the cytoplasm of neutrophils, released in the mucosa and the intestinal lumen leucocyte that can be detected in serum or body fluids as a potentially useful clinical inflammatory marker [55]. Elevated fecal calprotectin (FC) is used as an indicator of intestinal inflammation as it is released into the intestinal lumen during times of leukocyte shedding, cell disturbance, and cell death [56]. Toma et al. reported that the concentration of calprotectin is significantly increased in serum of patients infected with *G. lamblia* compared to the control group [57]. However, there were conflicting evidences on the utility of FC as a diagnostic biomarker for giardiasis as reports suggested that FC may not be significantly elevated in cases of acute, mild giardiasis, but may be elevated in persistent or severe giardiasis [58].

Another protein is Trefoil factor 3 (TFF3). TFF3 is a small peptide that plays an important role in mucosal protection, cell proliferation, and cell migration while the aberrant expression of TFF3 is associated with gastrointestinal inflammation, solid tumors, and other clinical diseases [59]. TFF3 was found in both in vitro and in vivo studies, leading to the hypothesis of its role in facilitating intestinal epithelial restitution, repair, and mucosal protection. In the intestine, TTF3 showed increased resistance to intestinal damage and ulceration that results from intestinal infection [60]. A study by Toma et al. showed a significant increase in the serum level of TFF3 in patients infected with *G. lamblia* parasite compared to the control group [57]. It is possible that the increase of TFF3 protein may be due to goblet cell mucin and the role of TFF3 in defending the intestinal mucosa from enteropathogens such as the *G. lamblia* parasite [61].

## 4. Molecular Biomarkers

Molecular diagnosis is not routinely performed to diagnose giardiasis, but it is rather an important procedure to distinguish the different subgenotypes of *Giardia* spp. *G. duodenalis* is classified into at least eight distinct genetic groups (A to H) or assemblages based on the comparisons of the electrophoretic mobility of enzymes and chromosomes. All these assemblages are morphologically identical and are indistinguishable by light microscopy, although molecular subtyping methods offer a high level of accuracy and specificity. Heyworth performed multilocus genotyping (MLG) based on the genetic loci of the small subunits of ribosomal RNA (ssu-rRNA), triose phosphate isomerase (tpi), glutamate dehydrogenase (gdh), and *β*-giardin (bg) genes, elongation factor 1-alpha (EF1*α*), GLORF-C4 (C4), and the intergenomic rRNA spacer region (IGS) to observe the prevalence of different assemblages and to find the correlation between genetic assemblages and clinical symptoms [62].

Loop-mediated isothermal amplification (LAMP) is another current method of gene amplification which employs six oligonucleotide primers designed based on the *G. lamblia* elongation factor 1 alpha (EF1*α*) gene sequence [63]. LAMP is considered to be field applicable because of its simple read-out method, which is observation through the naked eye. Other than that, multiplexing, real-time PCR, and high-resolution melting curve analysis (HRM) also offer prospects for multiple species and assemblage detection in automated procedures, in addition to being quantitative assays with increased sensitivity and specificity. Verweij et al. claimed that real-time PCR is as specific and sensitive as antigen detection and is more sensitive than microscopy when used to detect *G. lamblia* DNA in fecal samples [64]. As far as molecular biomarkers are concerned, a new microRNA-based method through deep-sequence profiling of *Giardia* small-RNA revealed the high expression of two miRNAs, namely miR5 and miR6, in *Giardia* trophozoites. The study further confirmed the presence of miR5 in duodenal biopsies of patients with *Giardia* infection, and this method seemed to be more sensitive when compared with testing for *Giardia* DNA by qPCR, hence suggesting that miR5 testing may be a new method for the diagnosis of giardiasis in patients undergoing endoscopy investigation for undiagnosed persistent abdominal symptoms [65].

## 5. Volatile Biomarkers

One of the other obvious criteria found in giardiasis patients with persistent diarrhea is the characteristic of feces with foul odor, which occurs as a result of the release of volatile organic compounds by the parasite. Central to this observation is the understanding that microbial metabolism releases various organic compounds that have unique smells, and when analyzed using gas chromatography, it was found that feces from *Clostridium difficile* patients produces 5-methyl-2-furancarboxyaldehyde [66] and isocaproic acid [67], feces from patients with *Campylobacter jejuni* presents 1-butoxy-2-propanol and 3-methyl furan [68], and feces from patients with *Vibrio cholerae* produces extremely large quantities of dimethyl disulphide and p-menth-1-en-8-ol [69].

In a previous study by Bond et al., the characteristic smell of the feces of giardiasis patients with persistent diarrhea was analyzed, resulting in the identification of several important compounds, namely, 2,2,4,4-tetramethyloctane, acetic acid, and 2,2,4,6,6-pentamethylheptane, suggesting that these were potential biomarkers of giardiasis [70]. Meanwhile, three possible biomarkers appear to be found in the feces of *Giardia* patients with chronic diarrhea, namely acetic acid, 1,4-dimethoxy-2,3-butanediol, and 1,3-dimethoxy-2-propanol [71]. It is worth noting that although gas chromatography may not sound feasible as a direct detection method for giardiasis, the detection of targeted volatile compounds may at least serve as an alternative method to be employed in other diagnostic formats for giardiasis.

## 6. Hormonal Biomarkers

Ghrelin is a hormone produced mainly by the stomach, and in small amounts released by the small intestine, pancreas, brain, kidney, myocardium, hypothalamus, and pituitary gland. Ghrelin is identified as an endogenous ligand for growth hormone secretagogue receptor [72]. Changes in ghrelin levels have been reported in cases of parasitic infections where the acylated ghrelin had been found to affect glucose metabolism by modulating insulin secretion, amino-acid uptake and bone formation, appetite, increased food intake, energy balance, gastrointestinal motility, cardiac performance, and anxiety [73]. A study by Al-Hadraawy et al. showed that serum ghrelin concentration in giardiasis patients was significantly lower than in the healthy group. This reduction is thought to offset the increase in glucose concentration and reduce the increased lipid peroxidation due parasitic infection [74].

Another hormone of interest is melatonin which is mainly secreted by the pineal gland, retina, gut, skin, platelets, bone marrow, and possibly other structures, of which systemic contribution is insignificant [75]. Melatonin can stimulate innate immune cells, primarily leukocytes, which represent an important anti-bacterial mechanism, yet little is known about its influence on protozoan infection. Al-Hadraawy et al. suggested that increased melatonin levels are a reflection to leukocytosis caused by *G. lamblia*, known as immunomodulator, based on the findings in which serum melatonin concentrations were significantly twice as high in giardiasis patients than in the healthy group [74]. Further studies are necessary to support whether hormonal and other generic biomarkers have the potential to be used as diagnostic and therapeutic biomarkers for giardiasis.  Table 1 summarizes the potential biomarkers associated with giardiasis, and the diagnostic strength of the associated biomarkers is illustrated in Table 2.

## 7. Conclusions

In diagnosing giardiasis, there is no single best method that can detect *Giardia* infection accurately. The sensitivity and specificity of the existing methods are influenced by the underlying factors inherent in the diagnosis technique, the skill of the personnel, and the intermittent nature of cyst excretion. Nonetheless, the advances in precision medicine and predictive diagnostics have paved the way for more discoveries to improve the accuracy of the diagnosis of giardiasis, for which biomarkers are one of the potential targets that warrant further study. Various biomarkers, including proteins, serological, inflammatory, molecular, volatile, and hormonal, are of potential diagnostic and therapeutic value for giardiasis; however, the most notable ones are VSPH7, VSP5G8, *α*-1-giardin, *α*- and *β*-tubulins, Hsp70, Hsp90, rCWP2, GSA 65, SIgA, and miR5. Some of these biomarkers have shown good potential and have already been applied in commercial testing. Clearly, pathogen-specific proteins are a more valuable indicator of the onset of giardiasis infection than host proteins because the latter are relatively generic biomarkers that are incapable of identifying a specific causative agent.

Early and accurate diagnosis is very important for the treatment and prevention of giardiasis: therefore, the study of these biomarkers could enable the development of better diagnostic and therapeutic methods, which ultimately benefit the treatment and management of patients infected with giardiasis.

## Figures and Tables

**Table 1:** The potential biomarkers associated with giardiasis.

No.	Group of Biomarkers	Biomarker	Source	Stage indicator	Assemblage	Origin of expression	Reference
1	*Proteins of Giardia* (i) Variant-specific surface proteins (VSPs)	VSP 5G8	Serum and fecal	Acute	B	Trophozoite	[20]
		VSPH7	Serum	Acute	B	Trophozoite	[76]
		Recombinant VSP3	Serum	Acute	A	Trophozoite	[12]
		Recombinant VSP5	Serum	Acute	B	Trophozoite	[12]
	(ii) Giardin	*α*-1-Giardin	Serum	Acute	A and B	Trophozoite and cyst	[25]
		*α*-7.1-Giardin and *α*-7.3 giardin	Serum	Acute	A	Trophozoite and cyst	[25]
		*α*-4-Giardin, *α*-5-giardin, *α*-6-giardin, *α*-8-giardin, *α*-9-giardin, *α*-10-giardin, *α*-11-giardin, *α*-12-giardin, *α*-13-giardin, *α*-15-giardin, *α*-16-giardin, *α*-17-giardin, *α*-18-giardin, and *α*-19-giardin	—	Acute	A and B	Trophozoite and cyst	[76]
		Recombinant *α*-1-giardin	Serum	Acute	A and B	Trophozoite and cyst	[25]
		Recombinant *α*-2-giardin	Serum	Acute	A and B	Trophozoite and cyst	[25]
		Recombinant *α*-7.1-giardin	Serum	Acute	A	Trophozoite and cyst	[25]
		Recombinant *α*-13-giardin	Serum	Acute	A	Trophozoite	[24]
	(iii) Tubulin	*α*-Tubulin	Serum	Acute	A and B	Trophozoite and cyst	[25]
		*β*-tubulin	Serum	Acute	A	Trophozoite and cyst	[25]
	(iv) Heat shock protein (Hsp)	Hsp 70	Serum	—	A and B	—	[35]
		Hsp 90	Serum	—	A	Trophozoite	[36]
	(v) Cyst wall	CWP 1	—	—	A and B	Cyst	[38]
		CWP 2	—	—	A and B	Cyst	[38]
		Recombinant CWP2	Fecal and serum	Chronic	A and B	Cyst	[39]
	(vi) Antigen	GSA 65	Fecal	Chronic	A	Trophozoite and cyst	[41]
	*Host-specific protein*	P-selectin	Serum	Chronic	—	—	[55]
		Calprotectin and Trefoil factor 3 (TFF3)	Serum	—	—	—	[58]
2	*Serological and other body fluids*	Immunoglobulin G (IgG)	Serum	Acute	—	Trophozoite	[43]
		Immunoglobulin M (IgM)	Serum	Acute	A	Trophozoite	[44]
		Immunoglobulin A (IgA)	Saliva and serum	—	A and B	Cyst	[45]
		Secretory IgA (SIgA) in saliva	Saliva	Acute	A	Trophozoite	[46]
		Secretory IgA (SIgA) in milk	Milk	Acute	A	Trophozoite	[48]
3	*Molecular*	miR5, miR6	Duodenal biopsies	Chronic	A	Trophozoite	[66]
4	*Volatile*	2,2,4,4-Tetramethyloctane, acetic acid and 2,2,4,6,6pentamethylheptane	Fecal	—	A and B	Trophozoite and cyst	[71]
		Acetic acid, 1,4-dimethoxy-2,3-butanediol, and 1,3-dimethoxy-2 propanol	Fecal	Chronic	—	Trophozoite and cyst	[72]
5	*Hormonal*	Ghrelin and melatonin	Serum	Acute	—	—	[75]

**Table 2:** Diagnostic strength of the identified biomarkers.

Type of test	Biomarker detected	Diagnostic strength	Strength/limitation in study	Reference
*Enzyme-linked immunosorbent assay (ELISA)*	Hsp70	The concentration of Hsp70 in fecal samples of patients with giardiasis was sevenfold higher than that in the control group	This study is the first to reveal the presence of Hsp70 in fecal matter of patients infected with *Giardia* parasites	[35]
	GSA 65	ELISA-GSA65 can detect giardiasis in at least 30% more cases than microscopy examination, with sensitivity and specificity ranging from 95% to 100%	Due to the lower level of antigen in the trophozoites that appear in the feces of infected patients, a very potent antiserum against the trophozoites that can detect such antigens was needed	[41]
	P-selectin	The level of P-selectin in male and female was 60.65 ± 3.42 and 77.52 ± 3.86, respectively, compared to that in the control group (42.3 ± 4.63 and 57.04 ± 4.79), respectively	—	[55]
	Calprotectin	The concentration of calprotectin in male (0.13907 ng/mL) and female patients (0.20530 ng/mL) infected with *G. lamblia* was significantly increased compared to that in the control group	—	[58]
	Trefoil factor 3 (TFF3)	The concentration of TFF3 in male (0.13907 ng/mL) and female patients (0.20530 ng/mL) infected with *G. lamblia* was significantly increased as compared to that in male (0.078864 ng/mL) and female (0.082629 ng/mL) patients in the control group	—	[58]
	Immunoglobulin G (IgG)	81% of 59 symptomatic giardiasis patients and 12% of 17 uninfected control subjects had circulating IgG antibodies to *G. lamblia*	The application of ELISA to screen large populations has been proven to be feasible and more sensitive than stool examination	[43]
	Immunoglobulin M (IgM)	Both sensitivity and specificity were 96%	Useful in identifying patients with current infection	[44]
	Secretory IgA (SIgA) in saliva	Levels of secretory anti-*Giardia* IgA showed better specificity (95%) than sensitivity (77%)	The advantage of assays from saliva is that it is simple and noninvasive, suitable for sampling especially in children	[45]
		Levels of secretory anti-*Giardia* IgA showed better specificity (94%) than sensitivity (74%)	The noninvasive nature of collecting samples of saliva represents a clear advantage for studies in children	[46]
	Secretory IgA (SIgA) in milk	Most milk and saliva samples contained anti-*Giardia* antibodies (59% and 52%, respectively), with a mean sIgA content 50 times higher in milk than in saliva	Since the function of antibodies in breast milk can bind to and inactivate secreted enzymes, further experiments are needed to show if this is important during infection	[48]
	Ghrelin	Serum ghrelin concentration in giardiasis patients (33.245 ng/mL) was significantly lower than that in healthy group (50.102 ng/mL)	The study included a relatively small number of male patients only. A larger number of patients and a more comprehensive study are recommended	[55]
	Melatonin	Serum melatonin concentration was higher in the giardiasis patients (22.876 pg/mL) than in the healthy group (9.213 pg/mL)	The study included a relatively small number of male patients only. A larger number of patients and a more comprehensive study are recommended	[55]
*Gas chromatography - mass spectrometry*	1. 2,2,4,4- tetramethyloctane, acetic acid and 2,2,4,6,6pentamethylheptane	These 3 VOCs had a significantly greater prevalence amongst *Giardia* cases (*p* < 0.0001). AUROC analysis demonstrated a value of 0.902	A study needs to be performed on a larger patient population so that severity of the infection can be better assessed and compared	[71]
	Acetic acid, 1,4-dimethoxy-2,3-butanediol and 1,3-dimethoxy-2-propanol,	The volatile alcohols 1,3-dimethoxy-2-propanol and 1,4-dimethoxy-2,3-butanediol and acetic acid have important weights in the separation of patients with giardiasis, showing high loadings of 0.809, 0.781, and 0.682	Analysis of volatile substances has sufficient sensitivity to detect differences in volatile profiles in the feces of patients with and without *G. duodenalis* infection	[72]
*Polymerase chain reaction (PCR)*	miR5 and miR6	The likelihood ratios for miR5 based on 100% sensitivity and specificity have a threshold cycle average < 33.5 while the likelihood ratios for miR6, based on 66.6% sensitivity and 90% specificity, have a threshold cycle average of 30.0	Relatively small numbers in both the experimental and control groups as well as assessment were limited to only two miRNA molecules	[66]
*Flow cytometry*	Recombinant VSP3 and recombinant VSP5	The sensitivity and specificity value of VSP5 and VSP3 on cumulative assays of IgG/IgM were 100% while the sensitivity and specificity value of IgA-VSP5 and VSP3 cumulative assays were 80% and 100%, respectively	The samples represent many *Giardia* strains as it examines the humoral immune response in giardiasis patients in nonendemic areas	[12]
	VSP 5G8	GS-5G8 (+) strain infection induced an antibody response that recognized more than 50% of the GS-5G8 (+) trophozoite population and (<90%) of GS-5G8 (+) during reinfection	Lack of knowledge for the variant shift of *G. lamblia* GS-5G8 (+) in culture or whether it has lost its ability to change to another VSP	[20]
